# Ultra-brief breath counting (mindfulness) training promotes recovery from stress-induced alcohol-seeking in student drinkers

**DOI:** 10.1016/j.addbeh.2019.106141

**Published:** 2020-03

**Authors:** Ruichong Shuai, Alexandra Elissavet Bakou, Lorna Hardy, Lee Hogarth

**Affiliations:** School of Psychology, University of Exeter, Washington Singer Building, Perry Road, Exeter EX4 4QG, UK

**Keywords:** Stress, Mood induction, Mindfulness, Breath counting, Alcohol choice

## Abstract

The therapeutic effect of mindfulness interventions on problematic drinking is thought to be driven by increased resilience to the impact of stress on negative mood and alcohol-seeking behaviour, but this claim needs empirical support. To address this hypothesis, the current study tested whether brief training of one component of mindfulness – breath counting – would reduce drinkers’ sensitivity to the effect of noise stress on subjective mood and alcohol-seeking behaviour. Baseline alcohol-seeking was measured by choice to view alcohol versus food thumbnail pictures in 192 student drinkers. Participants then received a 6-minute audio file which either trained breath counting or recited a popular science extract, in separate groups. All participants were then stressed by a loud industrial noise and alcohol-seeking was measured again simultaneously to quantify the change from baseline. Subjective mood was measured after all three stages (baseline, post intervention, post stress test). The breath counting group were instructed to deploy this technique during the stress test. Results showed that the breath counting versus control intervention improved subjective mood relative to baseline, attenuated the worsening of subjective mood produced by stress induction, and accelerated recovery from a stress induced increase in alcohol-seeking behaviour. Exploratory moderation analysis showed that this accelerated recovery from stress induced alcohol-seeking by breath counting was weaker in more alcohol dependent participants. Mindfulness therapies may improve problematic drinking by increasing resilience to stress induced negative mood and alcohol-seeking, as observed in this study. The weaker therapeutic effect of breath counting in more dependent drinkers may reveal limitations to this intervention strategy.

## Highlights

• Breath counting promoted recovery from stress induced alcohol-seeking.
• Breath counting attenuated stress induced changes in subjective negative affect.
• Breath counting might treat stress induced relapse.
• Mindfulness therapy may work via resilience to negative drinking triggers.

## 1 Introduction

Negative affective states play a major role in triggering problematic drinking and relapse (Baker, Piper, McCarthy, Majeskie, & Fiore, 2004). Prospective studies show that anxiety, depression, and self-reported drinking to cope with negative affect (coping motives) are prospective risk factors for the development/persistence of alcohol dependence and propensity to relapse (Boschloo et al., 2013, Bruce et al., 2005, Crum et al., 2008, Crum et al., 2013, Crum et al., 2013, Crum and Pratt, 2001, Gilman and Abraham, 2001, Holahan et al., 2001, Hussong et al., 2017, King et al., 2004, Kushner et al., 2005, Samet et al., 2013, Sihvola et al., 2008, Vernig and Orsillo, 2015, Zimmermann et al., 2003). Furthermore, the experimental induction of negative mood or stress increases alcohol motivation, and this effect is greater in those who report coping motives or depression symptoms (literature reviewed in: Hogarth et al., 2018, Hogarth et al., 2017). Finally, individual sensitivity to stress induced alcohol craving is associated with greater risk of relapse (Brady et al., 2006, Cooney et al., 1997, Higley et al., 2011, Sinha et al., 2011). Consequently, therapies have sought to build resilience to negative affect triggered alcohol motivation (Marlatt and Donovan, 2005, Stasiewicz et al., 2018).

Mindfulness therapies which train awareness and acceptance of negative emotions have reduced drinking frequency and relapse (Bowen et al., 2014, Garland and Howard, 2018, Li et al., 2017, Sancho et al., 2018, Stasiewicz et al., 2013, Zgierska et al., 2008; but see Grant et al., 2017), even if mindfulness training is relatively brief (Kamboj et al., 2017, Mermelstein and Garske, 2015; see also Tang, Tang, & Posner, 2013). Evidence that these therapeutic effects are mediated by increased resilience to negative affective drinking triggers comes from three studies. Witkiewitz, Bowen, Douglas, and Hsu (2013) found that the impact of mindfulness versus treatment as usual (TAU) on reduced alcohol craving was mediated by a latent variable that included acceptance of negative affect, but also included acting with awareness and nonjudgment, suggesting the mediator may be a complex construct. Hsu, Collins, and Marlatt (2013) found that the impact of mindfulness versus TAU on reduced alcohol use days was moderated such that individuals with lower distress tolerance benefited more, suggesting mindfulness may attenuate affective reactivity. Finally, Witkiewitz and Bowen (2010) reported a moderated mediation, wherein mindfulness therapy attenuated the mediational pathway between depression, craving and substance use, relative to TAU. These three studies support the claim that mindfulness interventions reduce drinking by building resilience to negative affective drinking triggers.

Trait mindfulness is similarly associated with resilience to negative affective drinking triggers in cross sectional studies with student drinkers. Bravo, Pearson, Stevens, and Henson (2016) reported a moderated mediation, wherein trait mindfulness predicted a weaker mediational pathway between depression and alcohol problems via self-reported drinking to cope with negative affect. Similar results have been reported by others (Roos et al., 2015, Bodenlos et al., 2013, Tull et al., 2015). Collectively, the foregoing studies suggest that experimentally trained (i.e. state) and trait mindfulness confer resilience to negative triggers for alcohol motivation, although the precise link between state and trait mindfulness remains unclear (Bravo, Pearson, Wilson, & Witkiewitz, 2018).

Importantly, emotional reactivity to negative mood and stress induction can be attenuated by extended mindfulness training (Basso et al., 2019, Brewer et al., 2009, Carroll and Lustyk, 2018, Crosswell et al., 2017, Hoge et al., 2013, Kral et al., 2018, Lillis et al., 2009, Ortner et al., 2007, Tang et al., 2007), and ultra-brief mindfulness training (8 and 20 min audio files or verbal script) (Adams et al., 2012, Carpenter et al., 2019, Liu et al., 2013, Masedo and Rosa Esteve, 2007, Sauer and Baer, 2012), although four ultra-brief studies have reported null effects (Evans et al., 2014, Luberto and McLeish, 2018, Paz et al., 2017, Vernig and Orsillo, 2009). It is worth noting that trait mindfulness also predicts reduced emotional reactivity to stress induction (Arch and Craske, 2010, Bullis et al., 2014), and greater self-reported distress tolerance/emotion regulation (Feldman et al., 2014, Feldman et al., 2007, Hsu et al., 2013, Luberto et al., 2014). In sum, emotional reactivity is clearly attenuated by longer mindfulness programs and trait mindfulness, but the effectiveness of ultra-brief mindfulness training remains equivocal.

The most important question is whether mindfulness interventions attenuate negative mood induced craving. There are three nominally positive studies. The first positive study found that 8 weeks of mindfulness based relapse prevention (MBRP) attenuated stress-induced alcohol/drug craving, compared to TAU, in substance dependent individuals (Carroll & Lustyk, 2018). The problem is that standard relapse prevention produced the same effect. Furthermore, in a separate study, cognitive-behavioral stress management also attenuated stress induced craving (Back, Gentilin, & Brady, 2007), so this effect is not specific to mindfulness interventions. The second positive study found, in treatment-seeking smokers, that mindfulness training versus psychoeducation attenuated neural stress reactivity measured by fMRI, and this predicted reduced smoking at follow up (Kober, Brewer, Height, & Sinha, 2017). However, the study did not test for an attenuation of stress-induced craving per se, so interpretation of the effect is ambiguous. The third positive study found in student drinkers that alcohol craving measured after stress induction was reduced by a subsequent 8-minute mindfulness versus educational audio, suggesting mindfulness promoted recovery from stress-induced craving (Bravo, Prince, O’Donnell, & Pearson, submitted for publication). However, because craving was not measured before stress induction, it is unclear whether mindfulness attenuated stress-induced craving as opposed to background craving.

These three studies need to be set against four null results. The first null study found in alcohol/cocaine abusers that although mindfulness versus CBT attenuated stress induced emotional reactivity, it did not attenuate stress-induced craving (Brewer et al., 2009). The second null study found in risky college drinkers that 10-min mindfulness versus relaxation training did not attenuate stress-induced changes in subjective mood or craving (Vinci et al., 2014). The third null study found in a group of daily smokers that 10-min guided mindful mediation versus popular science audio did not attenuate tobacco craving following stress induction (Luberto & McLeish, 2018). However, there was no stress induced increase in craving either, so the design was not optimal to test for an attenuation of this effect. The fourth null study produced a very similar pattern of results in female smokers (Adams et al., 2012). In sum, available studies are equivocal as to whether mindfulness training attenuates craving responses to negative triggers, and there is no obvious methodological parameter that distinguishes the positive from the negative findings.

To address these uncertainties, the current study tested whether one specific element of mindfulness therapy – breath counting (attention directed to breathing) – would attenuate stress-induced increases in alcohol-seeking behaviour, and subjective negative affect, measured in the lab. Breath counting was selected as the training manipulation because it is a core component of larger mindfulness packages, quickly engages attention to interoceptive states blocking out external distraction, it can be easily deployed in daily life by a wide range of groups making it practically useful, and breath counting accuracy correlates with trait mindfulness (Levinson et al., 2014, Wong et al., 2018). Importantly, briefly trained breath counting or mindful breathing techniques have been shown to attenuate or accelerate recovery from mood and stress induction effects on subjective mood (Arch and Craske, 2006, Goldin and Gross, 2010, Keng and Tan, 2018; see also: Feldman, Greeson, & Senville, 2010), improve cognitive performance (Gorman and Green, 2016, McHugh et al., 2010, Mrazek et al., 2012), and improve learning and problem solving (Kiken and Shook, 2011, McHugh et al., 2012, McHugh et al., 2010, Ramsburg and Youmans, 2012, Ramsburg and Youmans, 2014).

The current study tested whether breath counting would attenuate stress-induced increases in alcohol-seeking in undergraduate drinkers (n = 192). Baseline alcohol-seeking was first measured by preference to view alcohol versus food thumbnail pictures in a series of two-alternative forced choice trials. The pictorial choice measure has been well validated as an index of the relative value ascribed to drug versus food, and as a robust correlate of dependence symptom severity, drug use frequency, and other vulnerability markers such as coping motives and psychiatric symptoms in clinical and subclinical samples (Hardy et al., 2018, Hogarth and Hardy, 2018a, Moeller and Stoops, 2015). Participants then listened to a 6-minute audio file which either trained breath counting (the breath counting group), or recited an extract from a popular science book – Bill Bryson’s A Short History of Nearly Everything (the control group). All participants were then stressed by listening to a loud and unpleasant industrial noise (70 dB), during which alcohol choice was measured again, as at baseline, to measure the increase in alcohol-seeking (Cherek, 1985). The breath counting group were told to deploy the breath counting technique in the stress test. Subjective annoyance and happiness were measured at baseline, post intervention, and post stress test. It was predicted that in the control group stress induction would increase alcohol choice and annoyance and decrease happiness, and that these induction effects would be attenuated in the breath counting group. These data would support the hypothesis that mindfulness interventions achieve therapeutic impact on substance use outcomes by building resilience to acute stress triggers, and that brief breath counting training may have therapeutic potential in its own right.

## 2 Methods

### 2.1 Participants

192 participants, who had drunk at least once in the past month and were therefore not teetotal, were recruited from the University of Exeter student population (age range: 18–52 years) and were randomly assigned to either the breath counting group or control group. Participants provided informed consent, were debriefed and received a chocolate bar as the reimbursement for participation. The study was approved by the School of Psychology Research Ethics Committee.

### 2.2 Questionnaires

Participants completed the following questionnaires. The adult Patient-Reported Outcomes Measurement Information System Alcohol Use Short Form (PROMIS; Pilkonis et al., 2016) which contains 7 items assessing loss of control over drinking in the past 30 days (e.g., “I drank more than I planned”), endorsed on a 1–5 scale ranging from “Never” to “Always” (we report the average scale scores). The Alcohol Use Disorder Identification Test (AUDIT; Babor, Higgins-Biddle, Saunders, & Monteiro, 2001) which contains 10 items assessing the frequency of alcohol use and alcohol-related problems experienced in the past 12 months. Total scores can range from 0 to 40 split into categories: low-risk (0–7), hazardous (8–15), harmful (16–19) and possibly dependent (20–40). The modified five factor Drinking Motives Questionnaire Revised (DMQR; Grant, Stewart, O'Connor, Blackwell, & Conrod, 2007), which measures how frequently drinking is motived by each listed reason, on a 1–10 scale ranging from “Never” to “Almost always”. It has five subscales: drinking to cope with anxiety and depression, conformity, enhancement and socialising (the two coping subscales were collapsed). The Generalised Anxiety Disorder (GAD; Spitzer, Kroenke, Williams, & Lowe, 2006) scale which contains 7 items assessing generalised anxiety disorder in the past two weeks (e.g., “feeling nervous, anxious or on edge”). The score on each item ranges from 0 (“Not at all”) to 3 (“Nearly every day”). The total score can range from 0 to 21, with a score of 5, 10, and 15 as the cut-off points for mild, moderate and severe anxiety, respectively. The Patient Health Questionnaire depression scale (PHQ; Kroenke et al., 2009) which contains 8 items assessing depressive symptoms in the past two weeks (e.g., “little interest or pleasure in doing things”). The score on each item ranges from 0 (“Not at all”) to 3 (“Nearly every day”). The total score can range from 0 to 24, with a score of 5, 10, 15 and 20 as the cut-off points for mild, moderately severe and severe depression, respectively.

### 2.3 Procedure

#### 2.3.1 Baseline alcohol choice

**Fig. 1:**
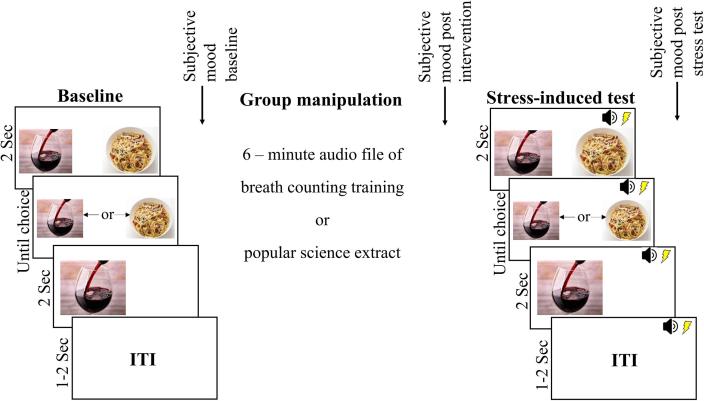
Task used to test whether breath counting promotes resilience to stress induced alcohol-seeking in student drinkers. Baseline alcohol choice was measured by preference to view alcohol versus food thumbnail pictures in two-alternative forced choice trials. Participants then listened to a six minute audio file which either trained breath counting or recited a popular audio book (control group). Alcohol choice was then measured again but with noise stress induction added. The breath counting group were told to deploy this technique during the stress test. Subjective happiness and annoyance were measured at the three timepoints denoted.

As shown in Fig. 1, alcohol pictorial choice was measured at baseline by participants completing 24 two-alternative forced-choice trials in which they freely chose to enlarge thumbnail pictures of either alcohol or food by pressing a left or right arrow key (Hardy & Hogarth, 2017). Instructions were: ‘In this task, you can view alcohol and food pictures by pressing the left or right arrow key’. In each trial, the alcohol and food thumbnail stimuli presented were each sampled from a set of 28 pictures, and presented randomly in the left or right screen position. The dependent variable was the percentage choice of alcohol across all choice trials. Following baseline alcohol choice, subjective mood was measured, at the baseline timepoint, by asking participants to what extent they currently felt happy and annoyed, in random order, on a 5-point scale ranging from 1 (“not at all”) to 5 (“extremely”).

#### 2.3.2 Breath counting versus control intervention

The half of participants who were assigned to the breath counting group listened to a 6-minute audio file (inspired by Ramsburg & Youmans, 2014) in which they were instructed (via a female voice) to relax and concentrate on their breath sensations, then count each outbreath, at normal pace, from one to ten, and then start again from one (see Supplementary material for full transcript and the audio file). The half of participants who were assigned to the control group received a 6-minute audio file in which was recited (by the same female voice as the breath counting audio) an extract from the popular science book A Short History of Nearly Everything by Bill Bryson (see Supplementary material). For both groups, after the audio file, participants were asked how much attention they had paid to the recording on a scale ranging from 1 (‘a little’) to 5 (‘a lot’), and how pleasant they had found to the experience on a scale ranging from 1 (‘Unpleasant’) to 5 (‘Pleasant’). Finally, all participants had their subjective happiness and annoyance measured at this post-intervention timepoint (identical to the baseline timepoint).

#### 2.3.3 The stress-induced alcohol choice test

All participants then completed an alcohol pictorial choice task identical to baseline, except a loud and unpleasant industrial noise (70 dB; file: airsander.mp3 from www.freesfx.co.uk) was played continuously through headphones over 36 trials, to induce mild stress and augment alcohol choice (Cherek, 1985). The 36 trials of the test phase were broken into three time bins of 12 trials each to examine changes over time. The breath counting group were instructed to deploy the breath counting technique during the stress test, whereas the control group received no comparable instruction. All participants reported their subjective happiness and annoyance identical to the baseline and post intervention timepoints. Finally, the breath counting group reported their attention to and pleasantness of the breath technique deployed during the stress test, identical to the post-intervention timepoint. In the end, all participants completed a mood repair procedure (Hardy & Hogarth, 2017) to normalise mood prior to departure (for ethical reasons).

#### 2.3.4 Analytical plan

ANOVAs were performed with the between subjects variable intervention group (breath counting, control) and the within subjects variable timepoint, which differed according to which dependent variable was considered. Percent alcohol choice was calculated from the baseline phase and the three time bins of the test phase, so progressive recovery from stress could be tested. Consequently, the block variable in this analysis had four levels: baseline and stress test bin 1–3. ANOVAs with subjective happiness and sadness included a timepoint variable with 3 levels (baseline, post intervention, post stress test). Pearson correlations were used to explore the relationship between questionnaire indices and behavioural/subjective measures in the task.

## 3 Results

The data that forms the basis of the results presented here are available from the University of Exeter Research Data Repository (https://ore.exeter.ac.uk/repository/), doi: TBC.

### 3.1 Participants

Four participants were excluded due to the extreme change in their percent alcohol picture choices from baseline to test that were greater than three times the interquartile range of the sample, leaving 188 participants for analysis. This did not change the pattern of significance of the results. As shown in Table 1, the breath counting and control groups were matched with respect to questionnaire measures. The breath counting group reported paying more attention to, and greater pleasantness of, the intervention at the post-intervention timepoint. Characterising the severity of alcohol use disorder symptoms in the sample as whole, the proportion of participants that fell into each AUDIT category were: low-risk (26%), hazardous (46%), harmful (18%), and possibly dependent (11%).

**Table 1:** Mean (SD, range) of questionnaire data reported by the breath counting and control groups. PROMIS = Patient-Reported Outcomes Measurement Information System Alcohol Use Short Form. AUDIT = Alcohol Use Disorder Identification Test. DMQR = modified Drinking Motives Questionnaire Revised. GAD = The Generalised Anxiety Disorder test. PHQ = Patient Health Questionnaire depression scale. *p* = significance level of the group contrast. – = test not possible.

	Group		*p*
	Breath counting (n = 93)	Control (n = 95)	
Age	21.51 (3.91, 18–52)	21.05 (2.09, 18–32)	0.32
Gender ratio (M/F)	47/46	48/47	1.00
PROMIS alcohol use	2.3 (0.8, 1–4.7)	2.4 (0.7, 1–4.1)	0.27
AUDIT score	11.44 (5.69, 2–28)	12.75 (6.16, 2–31)	0.13
DMQR coping	2.8 (1.8, 0–8.3)	3.2 (2.1, 0–9.2)	0.17
DMQR enhancement	4.9 (2.2, 0–9)	5.4 (2.2, 0–10)	0.11
DMQR socialising	6.7 (1.6, 1.4–9.8)	6.9 (1.8, 1.8–10)	0.43
DMQR conformity	1.6 (2.0, 0–9.2)	2.1 (1.9, 0–6.8)	0.10
GAD score	5.77 (4.51, 0–20)	6.79 (4.39, 0–21)	0.12
PHQ score	6.12 (4.74, 0–22)	6.65 (5.14, 0–24)	0.46
Attention to intervention (post-intervention)	3.98 (0.91, 1–5)	2.85 (1.15, 1–5)	<0.001
Pleasantness of intervention (post-intervention)	4.02 (0.92, 2–5)	3.01 (1.13, 1–5)	<0.001
Attention to intervention (post-test)	2.80 (1.15, 1–5)		–
Pleasantness of intervention (post-test)	2.98 (1.04, 1–5)		–

### 3.2 Subjective happiness

**Fig. 2:**
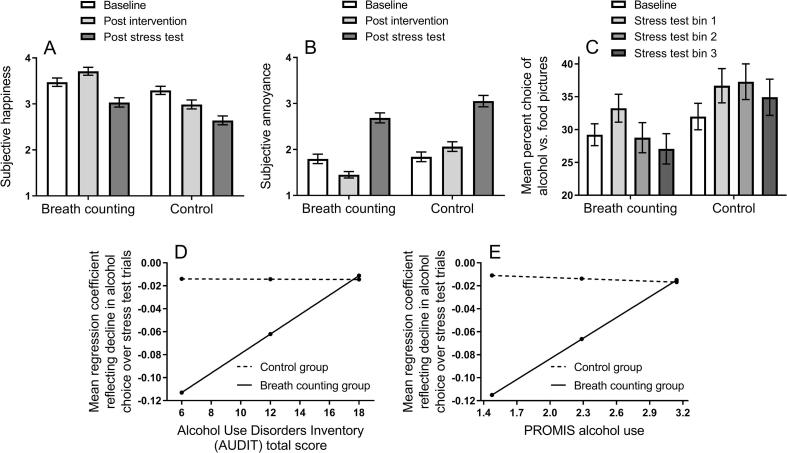
(A) Mean (and SEM) subjective happiness measured at three timepoints (baseline, post intervention and post stress test) in the two groups. The breath counting group relative to the control group showed greater happiness post intervention, and an attenuated stress induced decrease in happiness post stress test. (B) Mean subjective annoyance measured at three timepoints in the two groups. The breath counting group relative to the control group showed reduced annoyance post intervention, and an attenuated stress induced increase in annoyance post stress test. (C) Mean percent choice of alcohol versus food pictures at baseline and across three time bins of the stress test, in the breath counting and control group. Both groups showed an increase in alcohol choice at stress test bin 1 vs. baseline. The groups differed thereafter. In the breath counting group, alcohol choice declined linearly back to baseline across test bins 2 and 3. In the control group, alcohol choice was stable above baseline across the stress test. (D) Moderation analysis: the effect of breath counting versus control intervention on recovery from stress induced alcohol-seeking (i.e. decline in alcohol choice across stress test trials indexed by a regression coefficient), was moderated by AUDIT scores, such that breath counting produced steeper recovery in participants with low and intermediate, but not high, AUDIT scores. (E) Equivalent moderation analysis with PROMIS alcohol use scores. Breath counting produced steeper recovery from stress induced alcohol-seeking in participants with low and intermediate, but not high, PROMIS alcohol use scores.

Fig. 2A shows subjective happiness reported by the breath counting and control group at three timepoints of the experiment (baseline, post intervention, post stress test). ANOVA on these data yielded a significant main effect of timepoint, *F*(2,372) = 65.30, *p* < .000, η_p_^2^ = 0.260, suggesting that subjective happiness changed over time. There was also a significant interaction between intervention group and timepoint, *F*(2,372) = 12.98, *p* < .001, η_p_^2^ = 0.065, and a significant main effect of intervention group, *F*(1,186) = 13.39, *p* < .001, η_p_^2^ = 0.067, suggesting the intervention manipulation affected subjective happiness. Contrasts of the intervention groups indicated that their subjective happiness did not differ significantly at baseline, *F*(1,186) = 1.95, *p* = .16, η_p_^2^ = 0.010, but did differ significantly at post intervention, *F*(1,186) = 30.60, *p* < .001, η_p_^2^ = 0.141, and at post stress test, *F*(1,186) = 7.79, *p* = .006, η_p_^2^ = 0.040. Furthermore, contrasts of baseline versus post intervention timepoints indicated that breath counting significantly increased happiness, *F*(1,92) = 13.76, *p* < .001, η_p_^2^ = 0.130, whereas the control intervention decreased happiness, *F*(1,94) = 20.73, *p* < .001, η_p_^2^ = 0.181. These analyses suggest that breath counting compared to the control intervention increased happiness after the intervention, and protected from a stress induced decrease in happiness in the stress test.

### 3.3 Subjective annoyance

Fig. 2B shows subjective annoyance reported by the breath counting and control group at three timepoints of the experiment (baseline, post intervention, post stress test). ANOVA on these data yielded a significant main effect of timepoint, *F*(2,372) = 126.73*p* < .001, η_p_^2^ = 0.405, suggesting that subjective annoyance changed over time. There was also a significant interaction between intervention group and timepoint, *F*(2,372) = 6.50, *p* = .002, η_p_^2^ = 0.034, *p* < .000, η_p_^2^ = 0.405, and a significant main effect of intervention group, *F*(1,186) = 8.67, *p* = .004, η_p_^2^ = 0.045, suggesting the intervention manipulation affected subjective annoyance. Contrasts of the two intervention groups indicated that their subjective annoyance did not differ significantly at baseline, *F*(1,186) = 0.10, *p* = .73, η_p_^2^ = 0.001, but did differ significantly at the post intervention, *F*(1,186) = 23.24, *p* < .001, η_p_^2^ = 0.111, and at post stress test timepoints, *F*(1,186) = 4.91, *p* = .028, η_p_^2^ = 0.026. Furthermore, contrasts of baseline versus post intervention timepoints indicated that the breath counting intervention significantly decreased annoyance, *F*(1,92) = 17.78, *p* < .001, η_p_^2^ = 0.162, whereas the control intervention increased annoyance, *F*(1,94) = 5.05, *p* = .03, η_p_^2^ = 0.051. These analyses suggest that breath counting compared to the control intervention decreased annoyance after the intervention, and protected from a stress induced increase in annoyance in the stress test.

### 3.4 Percent alcohol picture choice

Fig. 2C shows percent alcohol picture choice measured at baseline and three time bins of the stress test for the breath counting and control group. ANOVA on these data yielded a significant interaction between intervention (2) and block (4), *F*(3,588) = 3.09, *p* = .027, η_p_^2^ = 0.016, suggesting that the breath counting group recovered more quickly from the stress induced alcohol-seeking effect. There was also a significant main effect of block, *F*(3,558) = 5.71, *p* = .001, η_p_^2^ = 0.030, but no effect of group, *F*(1,186) = 3.61, *p* = .059, η_p_^2^ = 0.019. Breakdown of the significant 2x4 interaction was achieved with a series of 2x2 ANOVAs. Analysis of baseline and test bin 1 yielded a significant main effect of block, *F*(1,186) = 13.88, *p* < .001, η_p_^2^ = 0.069, and no interaction between intervention group and block, *F*(1,186) = 0.08, *p* = .778, η_p_^2^ = 0.000, suggesting that stress increased alcohol choice at test bin 1 relative to baseline, and groups were matched in sensitivity to this effect (i.e. there was no immediate protective effect of breath counting). By contrast, analysis of baseline and test bin 2 again yielded a significant effect of block, *F*(1,186) = 4.59, *p* = .033, η_p_^2^ = 0.024, but also a significant interaction between intervention group and block, *F*(1,186) = 6.44, *p* = .012, η_p_^2^ = 0.033, suggesting that breath counting protected from stress induced alcohol-seeking at test bin 2. Similarly, analysis of baseline and test bin 3 yielded no significant effect of block, *F*(1,186) = 0.11, *p* = .741, η_p_^2^ = 0.001, and a significant interaction between intervention group by block, *F*(1,186) = 4.57, *p* = .034, η_p_^2^ = 0.024, again suggesting that breath counting protected from stress induced alcohol-seeking at test bin 3. One way ANOVAs comparing groups at each time bin found no significant difference at baseline, *F*(1,186) = 1.12, *p* = .292, η_p_^2^ = 0.006, or test bin 1, *F*(1,186) = 1.03, *p* = .311, η_p_^2^ = 0.006, but a significant difference at test bin 2, *F*(1,186) = 5.74, *p* = .018, η_p_^2^ = 0.030, and test bin 3, *F*(1,186) = 4.77, *p* = .030, η_p_^2^ = 0.025. Finally, examination of the three test bins indicated that there was a significant linear decline for the breath counting group, *F*(1,92) = 12.95, *p* = .001, η_p_^2^ = 0.123, but not the control group, *F*(1,94) = 1.04, *p* = .311, η_p_^2^ = 0.011. These analyses suggest that breath counting, compared to the control intervention, promoted recovery from a stress-induced increase in alcohol choice.

### 3.5 Exploratory correlations

Correlations tested whether, in the sample as a whole, baseline alcohol choice and the stress induced increase in alcohol choice were associated with questionnaire scales. Percent alcohol choice at baseline correlated significantly with AUDIT, *r* = 0.43, *p* < .001, PROMIS alcohol use, *r* = 0.34, *p* < .001, DMQR enhancement, *r* = 0.31, *p* < .001, DMQR social, *r* = 0.29, *p* < .001, and DMQR coping, *r* = 0.37, *p* < .001, but not DMQR conformity, *r* = 0.06, *p* = .34, GAD anxiety, *r* = 0.05, *p* = .52, or PHQ depression, *r* = 0.14, *p* = .06. The stress induced increase in alcohol choice from baseline to test (over all time bins) correlated positively with AUDIT, *r* = 0.16, *p* = .03, PROMIS alcohol use, *r* = 0.19, *p* = .008, and DMQR coping, *r* = 0.19, *p* = .007, but not with DMQR enhancement, *r* = 0.14, *p* = .06, DMQR social, *r* = 0.13, *p* = .08, DMQR conformity, *r* = 0.11, *p* = .14, GAD anxiety, *r* = 0.03, *p* = .69, or PHQ depression, *r* = 0.00, *p* = .99. These correlations suggest that baseline alcohol-seeking and stress induced alcohol-seeking are linked to a range of vulnerability factors.

### 3.6 Exploratory moderation analysis

Moderation analysis was used to test whether the beneficial effect of breath counting differed between individuals. Recovery from stress induced alcohol-seeking was quantified by calculating a regression slope for each participant relating the probability of choosing the alcohol picture over successive test trials. This recovery score was entered as the outcome variable, intervention group was entered as the predictor variable and each questionnaire was entered as the moderator, in separate moderation models using Hayes Process Software for SPSS (https://processmacro.org/index.html). A significant moderation effect was found for AUDIT, *b* = −0.01, *p* = .03, shown in Fig. 2D. The breath counting versus control intervention produced steeper recovery from stress induced alcohol-seeking in those with low, *b* = 0.09, *p* < .005, and intermediate AUDIT scores, *b* = 0.04, *p* < .05, but not those with high AUDIT scores, *b* = −0.00, *p* = .92. A significant moderation effect was also found with PROMIS alcohol use scores, *b* = −0.06, *p* = .04, shown in Fig. 2E. The breath counting versus control intervention produced steeper recovery from stress induced alcohol-seeking in those with low, *b* = 0.10, *p* < .005, and intermediate PROMIS alcohol use scores, *b* = 0.05, *p* = .03, but not those with high PROMIS alcohol use scores, *b* = −0.00, *p* = .95. Finally, there were no moderation effects with DMQR coping, *b* = 0.00, *p* = .97, DMQR social, *b* = −0.02, *p* = .09, DMQR enhancement, *b* = −0.01, *p* = .61, DMQR conformity, *b* = −0.00, *p* = .73, GAD anxiety, *b* = −0.00, *p* = .96, or PHQ depression, *b* = −0.00, *p* = .26. Finally, there were no significant moderation effects when the change in happiness or annoyance from baseline to test was entered as the outcome variable. The overall implication of these analyses is that breath counting produced less recovery from stress induced alcohol-seeking in those with greater alcohol dependence.

## 4 Discussion

The study found that following training of a breath counting technique (versus control), happiness increased and annoyance decreased, relative to baseline. Participants also rated the breath counting intervention as more pleasant and attention demanding, indicating this practice was pleasant and acceptable. Deployment of the breath counting technique during noise stress induction resulted in a smaller decrease in happiness and smaller increase in annoyance, relative to the control group, suggesting that breath counting attenuated stress induced negative mood. Finally, deployment of the breath counting technique during noise stress promoted more rapid recovery from the stress induced increase in alcohol-seeking over bins of the test block. These findings indicate that ultra-brief breath counting training can improve mood, and attenuate stress induced negative mood and alcohol motivation. The therapeutic impact of mindfulness interventions on problematic drinking could be driven by such stress resilience effects, and ultra-brief breath counting training could have therapeutic potential in its own right.

Breath counting attenuated stress induced negative mood, corroborating studies showing attenuation of mood/stress induced subjective/physiological responses by (a) mindfulness or meditation training (Basso et al., 2019, Brewer et al., 2009, Carroll and Lustyk, 2018, Hoge et al., 2013, Kral et al., 2018, Lillis et al., 2009, Ortner et al., 2007, Tang et al., 2007); (b) ultra-brief mindfulness interventions (Adams et al., 2012, Carpenter et al., 2019, Liu et al., 2013, Masedo and Rosa Esteve, 2007, Sauer and Baer, 2012); and (c) ultra-brief breath counting training similar to the one used here (Arch and Craske, 2006, Goldin and Gross, 2010, Keng and Tan, 2018). However, several mindfulness training studies have failed to demonstrate attenuation of emotional reactivity (Evans et al., 2014, Luberto and McLeish, 2018, Paz et al., 2017, Vernig and Orsillo, 2009). Nevertheless, the weight of evidence supports the idea that mindfulness/breath counting engenders resilience to mood/stress induced emotional responses. It remains unknown whether this mechanism plays a role in the therapeutic impact of mindfulness on drinking outcomes.

The novel contribution of the current study was to demonstrate that deployment of the briefly trained breath counting technique promoted recovery from a stress induced increase in alcohol choice across time bins of the stress test, compared to the control group. This finding corroborates three studies which have reported attenuation of mood/stress induced craving by mindfulness based relapse prevention (Carroll & Lustyk, 2018), cognitive-behavioral stress management (Back et al., 2007), and ultra-brief mindfulness training (Bravo et al., submitted for publication); for a potentially related fMRI effect see Kober et al. (2017). However, there remain four studies which have reported no impact of extended (Brewer et al., 2009) or ultra-brief mindfulness training (Adams et al., 2012, Luberto and McLeish, 2018, Vinci et al., 2014) on mood/stress induced craving. There are multiple methodological differences between the positive and negative studies. Therefore, the boundary conditions necessary to demonstrate attenuation of mood/stress induced drug motivation remain obscure. We can conclude that the current model is sensitive to this effect, making it attractive as an assay in future studies.

The current study cannot isolate the mechanism(s) by which the breath counting intervention created resilience to stress induced mood and alcohol-seeking. A wide range of behavioural manipulations have been shown to attenuate mood/stress induced effects on subjective mood or physiological reactivity. These include brief instructions about accepting emotions (Campbell-Sills et al., 2006, Keogh et al., 2005, Levitt et al., 2004, McMullen et al., 2008, Schartau et al., 2009, Singer and Dobson, 2007, Singer and Dobson, 2009, Vieten et al., 2010), guided imagery of the stressor prior to testing (Yaremko and Butler, 1975, Yaremko et al., 1972), guided positive mental imagery (Jacob et al., 2011), guided neutral mental imagery (Joormann and Siemer, 2004, Joormann et al., 2007), attentional capture by happy faces (Sanchez, Vazquez, Gomez, & Joormann, 2014), occupancy of working memory by secondary tasks (Erber and Tesser, 1992, Kron et al., 2010, Trask and Sigmon, 1999, Van Dillen et al., 2009, Van Dillen and Koole, 2007, Van Dillen and Koole, 2009), distress tolerance training (Bornovalova, Gratz, Daughters, Hunt, & Lejuez, 2012), acute exercise (Bernstein and McNally, 2017a, Bernstein and McNally, 2017b, Mata et al., 2013, Rejeski et al., 1992), exposure to green environments (Jiang, Li, Larsen, & Sullivan, 2014) and drawing pleasant pictures (Dalebroux et al., 2008, Drake et al., 2011, Drake and Winner, 2012, Smolarski et al., 2015). Although any of these processes could be responsible for the effects found in the present study, occupancy of working memory resources is perhaps the most plausible mechanism (Tapper, 2018). If, over the stress test, participants became more skilled in attending to the breath counting exercise, diverting attention from the noise, this would explain the progressive recovery from stress induced alcohol-seeking. Future studies need to isolate the effective mechanism by including an active control group (e.g. number counting, relaxation training), and by inserting measures (e.g. state mindfulness, breath counting accuracy) to test mediation of the therapeutic outcome.

There were also individual differences in the observed effects. First, baseline alcohol choice was associated with multiple indices of vulnerability to alcohol dependence (AUDIT, PROMIS alcohol use, DMQR subscales), as has been reported previously in clinical and subclinical samples (Hardy and Hogarth, 2017, Hardy et al., 2018, Hogarth and Hardy, 2018b, Hogarth et al., 2018), consistent with the relative value of alcohol playing a role in dependence risk. Interestingly, percent alcohol choice did not correlate with anxiety and depression, confirming similar null associations with student drinkers (Hogarth and Hardy, 2018b, Hogarth et al., 2018), and contradicting studies with more severe drinker samples (Hardy and Hogarth, 2017, Hardy et al., 2018). The implication is that psychiatric symptoms play a more powerful role in alcohol valuation in more severe drinkers.

Second, the magnitude of the stress induced increase in alcohol-seeking from baseline to test, in the sample as a whole, increased with AUDIT and PROMIS alcohol use measures. By contrast, three previous studies found that AUDIT was not associated with negative mood induced alcohol-seeking (Hardy and Hogarth, 2017, Hogarth and Hardy, 2018b, Hogarth et al., 2018), suggesting stress induction may favour this association. However, in the wider literature, the association between dependence and mood/stress induced craving is inconsistent (Austin and Smith, 2008, Cooney et al., 1997, Field and Powell, 2007, Field and Quigley, 2009, Randall and Cox, 2001, Sinha et al., 2009, Woud et al., 2015, Zack et al., 2003, Zack et al., 2006, Zack et al., 1999), although the link to relapse risk is more reliable (Brady et al., 2006, Cooney et al., 1997, Higley et al., 2011, Sinha et al., 2011). The implication is that stress/mood induced drug motivation may increase with only particular dimensions of dependence, yet to be clarified.

Third, the stress induced increase in alcohol-seeking correlated with DMQR coping, but not other DMQR subscales. Similar selective associations have been reported in other studies (Austin and Smith, 2008, Birch et al., 2004, Field and Powell, 2007, Field and Quigley, 2009; Grant et al., 2007, Hogarth and Hardy, 2018b, Hogarth et al., 2019, Hogarth et al., 2018, Rousseau et al., 2011, Woud et al., 2015). By contrast, stress induced alcohol-seeking did not correlate with anxiety or depression symptoms, which contradicts findings from both student and clinical samples (Cooney et al., 1997, Fucito and Juliano, 2009, Hogarth et al., 2018, Hogarth et al., 2017). One explanation is that although psychiatric symptoms confer sensitivity to negative affect drug use triggers, this relationship is proximally mediated by coping motives (Cox and Klinger, 1988, Hogarth et al., 2019), such that the correlation between psychiatric symptoms and mood/stress induced drug-seeking is weaker and more unreliable. However, this model requires empirical confirmation.

Finally, the moderation analyses showed that breath counting produced less recovery from stress induced alcohol-seeking as dependence severity increased, and produced no recovery in more dependent drinkers. It is possible that this resistance to recovery was a corollary of more dependent drinkers showing a greater stress induced alcohol-seeking effect overall. In any case, the implication is that breath counting (and by extension mindfulness training) may be less effective for more dependent drug users. However, this claim contradicts a moderation analysis of clinical trials data which showed that mindfulness based relapse prevention versus control interventions produced a bigger effect on substance use outcomes as dependence symptom severity increased (Roos, Bowen, & Witkiewitz, 2017), i.e. greater efficacy for more dependent individuals. It is difficult to resolve the discrepancy between these findings. It is possible that more dependent individuals benefit more from extended interventions, and less from brief interventions, or there may be unique facets of mindfulness training not encompassed by breath counting. Regardless, the findings from the present study demand that the effect of breath counting on recovery from stress induced alcohol-seeking is tested in more severe drinkers to assess whether this approach has therapeutic potential in a non-student sample.

To conclude, the study found that a briefly trained breath counting technique improved mood, attenuated stress induced worsening of mood, and promoted recovery from stress induced alcohol-seeking in student drinkers. Mindfulness therapies may improve drinking outcomes via these effects. This possibility could be evaluated in a mindfulness clinical trial by testing whether the treatment effect on drinking outcomes is mediated by an effect on stress induced subjective mood and alcohol-seeking (Hsu et al., 2013, Magill and Longabaugh, 2013, Witkiewitz and Bowen, 2010, Witkiewitz et al., 2013). The second conclusion is that brief breath counting training might have therapeutic potential in its own right. However, this claim is limited because breath counting did not promote recovery from stress induced alcohol-seeking in more dependent drinkers. The clinical potential of breath counting needs to be evaluated in a more severe drinker sample. Finally, the finding that stress induced alcohol-seeking was sensitive to the breath counting intervention suggests this model could be used to screen other candidate interventions designed to mitigate this effect, such as anxiolytic pharmacotherapy (Mantsch et al., 2016, Schwandt et al., 2016, Spanagel et al., 2014).

## Role of Funding Sources

The research was supported by an Alcohol Change grant (RS17/03) and a Medical Research Council (UK, MRC) Confidence in Global Mental Health pump priming award (MC_PC_MR/R019991/1) to Hogarth. Funders had no role in the study design, collection, analysis or interpretation of the data, writing the manuscript, or the decision to submit the paper for publication.

## Contributors

Hogarth, Hardy and Bakou designed the study. Bakou implemented the design and created materials. Shuai ran the study and wrote the first draft of the paper. Hogarth conducted the analysis. All authors contributed to the final article.

## Declaration of Competing Interest

The authors declare that they have no known competing financial interests or personal relationships that could have appeared to influence the work reported in this paper.

## References

[b0005] Adams C.E., Benitez L., Kinsaul J., Apperson McVay M., Barbry A., Thibodeaux A., Copeland A.L. (2012). Effects of brief mindfulness instructions on reactions to body image stimuli among female smokers: An experimental study. Nicotine & Tobacco Research.

[b0010] Arch J.J., Craske M.G. (2006). Mechanisms of mindfulness: Emotion regulation following a focused breathing induction. Behaviour Research and Therapy.

[b0015] Arch J.J., Craske M.G. (2010). Laboratory stressors in clinically anxious and non-anxious individuals: The moderating role of mindfulness. Behaviour Research and Therapy.

[b0020] Austin J.L., Smith J.E. (2008). Drinking for negative reinforcement: The semantic priming of alcohol concepts. Addictive Behaviors.

[b0025] Babor T.F., Higgins-Biddle J.C., Saunders J.B., Monteiro M.G. (2001). AUDIT: The alcohol use disorders identification test guidelines for use in primary care.

[b0030] Back S.E., Gentilin S., Brady K.T. (2007). Cognitive-behavioral stress management for individuals with substance use disorders: A pilot study. The Journal of Nervous and Mental Disease.

[b0035] Baker T.B., Piper M.E., McCarthy D.E., Majeskie M.R., Fiore M.C. (2004). Addiction motivation reformulated: An affective processing model of negative reinforcement. Psychological Review.

[b0040] Basso J.C., McHale A., Ende V., Oberlin D.J., Suzuki W.A. (2019). Brief, daily meditation enhances attention, memory, mood, and emotional regulation in non-experienced meditators. Behavioural Brain Research.

[b0045] Bernstein E.E., McNally R.J. (2017). Acute aerobic exercise hastens emotional recovery from a subsequent stressor. Health Psychology.

[b0050] Bernstein E.E., McNally R.J. (2017). Acute aerobic exercise helps overcome emotion regulation deficits. Cognition and Emotion.

[b0055] Birch C.D., Stewart S.H., Wall A., McKee S.A., Eisnor S.J., Theakston J.A. (2004). Mood-induced increases in alcohol expectancy strength in internally motivated drinkers. Psychology of Addictive Behaviors.

[b0060] Bodenlos J.S., Noonan M., Wells S.Y. (2013). Mindfulness and alcohol problems in college students: The mediating effects of stress. Journal of American College Health.

[b0065] Bornovalova M.A., Gratz K.L., Daughters S.B., Hunt E.D., Lejuez C.W. (2012). Initial RCT of a distress tolerance treatment for individuals with substance use disorders. Drug and Alcohol Dependence.

[b0070] Boschloo L., Vogelzangs N., van den Brink W., Smit J.H., Veltman D.J., Beekman A.T., Penninx B.W. (2013). Depressive and anxiety disorders predicting first incidence of alcohol use disorders: Results of the Netherlands Study of Depression and Anxiety (NESDA). Journal of Clinical Psychiatry.

[b0075] Bowen S., Witkiewitz K., Clifasefi S.L., Grow J., Chawla N., Hsu S.H., Larimer M.E. (2014). Relative efficacy of mindfulness-based relapse prevention, standard relapse prevention, and treatment as usual for substance use disorders: A randomized clinical trial. JAMA Psychiatry.

[b0080] Brady K.T., Back S.E., Waldrop A.E., McRae A.L., Anton R.F., Upadhyaya H.P., Randall P.K. (2006). Cold pressor task reactivity: Predictors of alcohol use among alcohol-dependent individuals with and without comorbid posttraumatic stress disorder. Alcoholism: Clinical and Experimental Research.

[b0085] Bravo, A. J., Prince, M. A., O’Donnell, M. B., Pearson, M. R., & Henson, M. M. (submitted for publication). Buffering the associations between negative mood states and subjective alcohol craving: A brief mindfulness induction. Addictive Behaviors.

[b0090] Bravo A.J., Pearson M.R., Stevens L.E., Henson J.M. (2016). Depressive symptoms and alcohol-related problems among college students: A moderated-mediated model of mindfulness and drinking to cope. Journal of Studies on Alcohol and Drugs.

[b0095] Bravo A.J., Pearson M.R., Wilson A.D., Witkiewitz K. (2018). When traits match states: Examining the associations between self-report trait and state mindfulness following a state mindfulness induction. Mindfulness.

[b0100] Brewer J.A., Sinha R., Chen J.A., Michalsen R.N., Babuscio T.A., Nich C., Rounsaville B.J. (2009). Mindfulness training and stress reactivity in substance abuse: Results from a randomized, controlled stage I pilot study. Substance Abuse.

[b0105] Bruce S.E., Yonkers K.A., Otto M.W., Eisen J.L., Weisberg R.B., Pagano M., Keller M.B. (2005). Influence of psychiatric comorbidity on recovery and recurrence in generalized anxiety disorder, social phobia, and panic disorder: A 12-year prospective study. American Journal of Psychiatry.

[b0110] Bullis J.R., Bøe H.J., Asnaani A., Hofmann S.G. (2014). The benefits of being mindful: Trait mindfulness predicts less stress reactivity to suppression. Journal of Behavior Therapy and Experimental Psychiatry.

[b0115] Campbell-Sills L., Barlow D.H., Brown T.A., Hofmann S.G. (2006). Effects of suppression and acceptance on emotional responses of individuals with anxiety and mood disorders. Behaviour Research and Therapy.

[b0120] Carpenter J.K., Sanford J., Hofmann S.G. (2019). The effect of a brief mindfulness training on distress tolerance and stress reactivity. Behavior Therapy.

[b0125] Carroll H., Lustyk M.K.B. (2018). Mindfulness-based relapse prevention for substance use disorders: Effects on cardiac vagal control and craving under stress. Mindfulness.

[b0130] Cherek D.R. (1985). Effects of acute exposure to increased levels of background industrial noise on cigarette smoking behavior. International Archives of Occupational and Environmental Health.

[b0135] Cooney N.L., Litt M.D., Morse P.A., Bauer L.O., Gaupp L. (1997). Alcohol cue reactivity, negative-mood reactivity, and relapse in treated alcoholic men. Journal of Abnormal Psychology.

[b0140] Cox W.M., Klinger E. (1988). A motivational model of alcohol use. Journal of Abnormal Psychology.

[b0145] Crosswell A.D., Moreno P.I., Raposa E.B., Motivala S.J., Stanton A.L., Ganz P.A., Bower J.E. (2017). Effects of mindfulness training on emotional and physiologic recovery from induced negative affect. Psychoneuroendocrinology.

[b0150] Crum R.M., Green K.M., Storr C.L., Chan Y.F., Ialongo N., Stuart E.A., Anthony J.C. (2008). Depressed mood in childhood and subsequent alcohol use through adolescence and young adulthood. Archives of General Psychiatry.

[b0155] Crum R.M., La Flair L., Storr C.L., Green K.M., Stuart E.A., Alvanzo A.A.H., Mojtabai R. (2013). Reports of drinking to self-medicate anxiety symptoms: Longitudinal assessment for subgroups of individuals with alcohol dependence. Depression and Anxiety.

[b0160] Crum R.M., Mojtabai R., Lazareck S., Bolton J.M., Robinson J., Sareen J., Storr C.L. (2013). A prospective assessment of reports of drinking to self-medicate mood symptoms with the incidence and persistence of alcohol dependence. JAMA Psychiatry.

[b0165] Crum R.M., Pratt L.A. (2001). Risk of heavy drinking and alcohol use disorders in social phobia: A prospective analysis. American Journal of Psychiatry.

[b0170] Dalebroux A., Goldstein T.R., Winner E. (2008). Short-term mood repair through art-making: Positive emotion is more effective than venting. Motivation and Emotion.

[b0175] Drake J.E., Coleman K., Winner E. (2011). Short-term mood repair through art: Effects of medium and strategy. Art Therapy.

[b0180] Drake J.E., Winner E. (2012). Confronting sadness through art-making: Distraction is more beneficial than venting. Psychology of Aesthetics, Creativity, and the Arts.

[b0185] Erber R., Tesser A. (1992). Task effort and the regulation of mood: The absorption hypothesis. Journal of Experimental Social Psychology.

[b0190] Evans D.R., Eisenlohr-Moul T.A., Button D.F., Baer R.A., Segerstrom S.C. (2014). Self-regulatory deficits associated with unpracticed mindfulness strategies for coping with acute pain. Journal of Applied Social Psychology.

[b0195] Feldman G., Dunn E., Stemke C., Bell K., Greeson J. (2014). Mindfulness and rumination as predictors of persistence with a distress tolerance task. Personality and Individual Differences.

[b0200] Feldman G., Greeson J., Senville J. (2010). Differential effects of mindful breathing, progressive muscle relaxation, and loving-kindness meditation on decentering and negative reactions to repetitive thoughts. Behaviour Research and Therapy.

[b0205] Feldman G., Hayes A., Kumar S., Greeson J., Laurenceau J.-P. (2007). Mindfulness and emotion regulation: The development and initial validation of the cognitive and affective mindfulness scale-revised (CAMS-R). Journal of Psychopathology and Behavioral Assessment.

[b0210] Field M., Powell H. (2007). Stress increases attentional bias for alcohol cues in social drinkers who drink to cope. Alcohol and Alcoholism.

[b0215] Field M., Quigley M. (2009). Mild stress increases attentional bias in social drinkers who drink to cope: A replication and extension. Experimental and Clinical Psychopharmacology.

[b0220] Fucito L.M., Juliano L.M. (2009). Depression moderates smoking behavior in response to a sad mood induction. Psychology of Addictive Behaviors.

[b0225] Garland E.L., Howard M.O. (2018). Mindfulness-based treatment of addiction: Current state of the field and envisioning the next wave of research. Addiction Science & Clinical Practice.

[b0230] Gilman S.E., Abraham H.D. (2001). A longitudinal study of the order of onset of alcohol dependence and major depression. Drug and Alcohol Dependence.

[b0235] Goldin P.R., Gross J.J. (2010). Effects of mindfulness-based stress reduction (MBSR) on emotion regulation in social anxiety disorder. Emotion.

[b0240] Gorman T.E., Green C.S. (2016). Short-term mindfulness intervention reduces the negative attentional effects associated with heavy media multitasking. Scientific Reports.

[b0245] Grant S., Colaiaco B., Motala A., Shanman R., Booth M., Sorbero M., Hempel S. (2017). Mindfulness-based relapse prevention for substance use disorders: A systematic review and meta-analysis. Journal of Addiction Medicine.

[b0250] Grant V.V., Stewart S.H., Birch C.D. (2007). Impact of positive and anxious mood on implicit alcohol-related cognitions in internally motivated undergraduate drinkers. Addictive Behaviors.

[b0255] Grant V.V., Stewart S.H., O'Connor R.M., Blackwell E., Conrod P.J. (2007). Psychometric evaluation of the five-factor Modified Drinking Motives Questionnaire — Revised in undergraduates. Addictive Behaviors.

[b0260] Hardy L., Hogarth L. (2017). A novel concurrent pictorial choice model of mood-induced relapse in hazardous drinkers. Experimental and Clinical Psychopharmacology.

[b0265] Hardy L., Parker S., Hartley L., Hogarth L. (2018). A concurrent pictorial drug choice task marks multiple risk factors in treatment-engaged smokers and drinkers. Behavioural Pharmacology.

[b0270] Higley A., Crane N., Spadoni A., Quello S., Goodell V., Mason B. (2011). Craving in response to stress induction in a human laboratory paradigm predicts treatment outcome in alcohol-dependent individuals. Psychopharmacology (Berl).

[b0275] Hogarth L., Hardy L. (2018). Alcohol dependence symptoms are associated with greater relative value ascribed to alcohol, but not greater discounting of costs imposed on alcohol. Psychopharmacology (Berl).

[b0280] Hogarth L., Hardy L. (2018). Depressive statements prime goal-directed alcohol-seeking in individuals who report drinking to cope with negative affect. Psychopharmacology (Berl).

[b0285] Hogarth L., Hardy L., Bakou A.E., Mahlberg J., Weidemann G., Cashel S., Moustafa A.A. (2019). Negative mood induction increases choice of heroin versus food pictures in opiate dependent individuals: correlation with self-medication coping motives and subjective reactivity. Frontiers in Psychiatry.

[b0290] Hogarth L., Hardy L., Mathew A.R., Hitsman B. (2018). Negative mood-induced alcohol-seeking is greater in young adults who report depression symptoms, drinking to cope, and subjective reactivity. Experimental and Clinical Psychopharmacology.

[b0295] Hogarth L., Martin L., Seedat S. (2019). Relationship between childhood abuse and substance misuse problems is mediated by substance use coping motives, in school attending South African adolescents. Drug and Alcohol Dependence.

[b0300] Hogarth L., Mathew A.R., Hitsman B. (2017). Current major depression is associated with greater sensitivity to the motivational effect of both negative mood induction and abstinence on tobacco-seeking behavior. Drug and Alcohol Dependence.

[b0305] Hoge E.A., Bui E., Marques L., Metcalf C.A., Morris L.K., Robinaugh D.J., Simon N.M. (2013). Randomized controlled trial of mindfulness meditation for generalized anxiety disorder: Effects on anxiety and stress reactivity. The Journal of Clinical Psychiatry.

[b0310] Holahan C.J., Moos R.H., Holahan C.K., Cronkite R.C., Randall P.K. (2001). Drinking to cope, emotional distress and alcohol use and abuse: A ten-year model. Journal of Studies on Alcohol.

[b0315] Hsu S.H., Collins S.E., Marlatt G.A. (2013). Examining psychometric properties of distress tolerance and its moderation of mindfulness-based relapse prevention effects on alcohol and other drug use outcomes. Addictive Behaviors.

[b0320] Hussong A.M., Ennett S.T., Cox M.J., Haroon M. (2017). A systematic review of the unique prospective association of negative affect symptoms and adolescent substance use controlling for externalizing symptoms. Psychology of Addictive Behaviors.

[b0325] Jacob G.A., Arendt J., Kolley L., Scheel C.N., Bader K., Lieb K., Tüscher O. (2011). Comparison of different strategies to decrease negative affect and increase positive affect in women with borderline personality disorder. Behaviour Research and Therapy.

[b0330] Jiang B., Li D., Larsen L., Sullivan W.C. (2014). A dose-response curve describing the relationship between urban tree cover density and self-reported stress recovery. Environment and Behavior.

[b0335] Joormann J., Siemer M. (2004). Memory accessibility, mood regulation, and dysphoria: Difficulties in repairing sad mood with happy memories?. Journal of Abnormal Psychology.

[b0340] Joormann J., Siemer M., Gotlib I.H. (2007). Mood regulation in depression: Differential effects of distraction and recall of happy memories on sad mood. Journal of Abnormal Psychology.

[b0345] Kamboj S.K., Irez D., Serfaty S., Thomas E., Das R.K., Freeman T.P. (2017). Ultra-brief mindfulness training reduces alcohol consumption in at-risk drinkers: A randomized double-blind active-controlled experiment. International Journal of Neuropsychopharmacology.

[b0350] Keng S.-L., Tan H.H. (2018). Effects of brief mindfulness and loving-kindness meditation inductions on emotional and behavioral responses to social rejection among individuals with high borderline personality traits. Behaviour Research and Therapy.

[b0355] Keogh E., Bond F.W., Hanmer R., Tilston J. (2005). Comparing acceptance- and control-based coping instructions on the cold-pressor pain experiences of healthy men and women. European Journal of Pain.

[b0360] Kiken L.G., Shook N.J. (2011). Looking up: Mindfulness increases positive judgments and reduces negativity bias. Social Psychological and Personality Science.

[b0365] King S.M., Iacono W.G., McGue M. (2004). Childhood externalizing and internalizing psychopathology in the prediction of early substance use. Addiction.

[b0370] Kober H., Brewer J.A., Height K.L., Sinha R. (2017). Neural stress reactivity relates to smoking outcomes and differentiates between mindfulness and cognitive-behavioral treatments. NeuroImage.

[b0375] Kral T.R.A., Schuyler B.S., Mumford J.A., Rosenkranz M.A., Lutz A., Davidson R.J. (2018). Impact of short- and long-term mindfulness meditation training on amygdala reactivity to emotional stimuli. NeuroImage.

[b0380] Kroenke K., Strine T.W., Spitzer R.L., Williams J.B., Berry J.T., Mokdad A.H. (2009). The PHQ-8 as a measure of current depression in the general population. Journal of Affective Disorders.

[b0385] Kron A., Schul Y., Cohen A., Hassin R.R. (2010). Feelings don't come easy: Studies on the effortful nature of feelings. Journal of Experimental Psychology: General.

[b0390] Kushner M.G., Abrams K., Thuras P., Hanson K.L., Brekke M., Sletten S. (2005). Follow-up study of anxiety disorder and alcohol dependence in comorbid alcoholism treatment patients. Alcoholism: Clinical and Experimental Research.

[b0395] Levinson D.B., Stoll E.L., Kindy S.D., Merry H.L., Davidson R.J. (2014). A mind you can count on: Validating breath counting as a behavioral measure of mindfulness. Frontiers in Psychology.

[b0400] Levitt J.T., Brown T.A., Orsillo S.M., Barlow D.H. (2004). The effects of acceptance versus suppression of emotion on subjective and psychophysiological response to carbon dioxide challenge in patients with panic disorder. Behavior Therapy.

[b0405] Li W., Howard M.O., Garland E.L., McGovern P., Lazar M. (2017). Mindfulness treatment for substance misuse: A systematic review and meta-analysis. Journal of Substance Abuse Treatment.

[b0410] Lillis J., Hayes S.C., Bunting K., Masuda A. (2009). Teaching acceptance and mindfulness to improve the lives of the obese: A preliminary test of a theoretical model. Annals of Behavioral Medicine.

[b0415] Liu X., Wang S., Chang S., Chen W., Si M. (2013). Effect of brief mindfulness intervention on tolerance and distress of pain induced by cold-pressor task. Stress and Health.

[b0420] Luberto C.M., McLeish A.C. (2018). The effects of a brief mindfulness exercise on state mindfulness and affective outcomes among adult daily smokers. Addictive Behaviors.

[b0425] Luberto C.M., McLeish A.C., Robertson S.A., Avallone K.M., Kraemer K.M., Jeffries E.R. (2014). The role of mindfulness skills in terms of distress tolerance: A pilot test among adult daily smokers. The American Journal on Addictions.

[b0430] Magill M., Longabaugh R. (2013). Efficacy combined with specified ingredients: A new direction for empirically supported addiction treatment. Addiction.

[b0435] Mantsch J.R., Baker D.A., Funk D., Le A.D., Shaham Y. (2016). Stress-induced reinstatement of drug seeking: 20 years of progress. Neuropsychopharmacology.

[b0440] Marlatt G.A., Donovan D.M. (2005). Relapse Prevention: Maintenance Strategies in the Treatment of Addictive Behaviors.

[b0445] Masedo A.I., Rosa Esteve M. (2007). Effects of suppression, acceptance and spontaneous coping on pain tolerance, pain intensity and distress. Behaviour Research and Therapy.

[b0450] Mata J., Hogan C.L., Joormann J., Waugh C.E., Gotlib I.H. (2013). Acute exercise attenuates negative affect following repeated sad mood inductions in persons who have recovered from depression. Journal of Abnormal Psychology.

[b0455] McHugh L., Procter J., Herzog M., Schock A.-K., Reed P. (2012). The effect of mindfulness on extinction and behavioral resurgence. Learning & Behavior.

[b0460] McHugh L., Simpson A., Reed P. (2010). Mindfulness as a potential intervention for stimulus over-selectivity in older adults. Research in Developmental Disabilities.

[b0465] McMullen J., Barnes-Holmes D., Barnes-Holmes Y., Stewart I., Luciano C., Cochrane A. (2008). Acceptance versus distraction: Brief instructions, metaphors and exercises in increasing tolerance for self-delivered electric shocks. Behaviour Research and Therapy.

[b0470] Mermelstein L.C., Garske J.P. (2015). A brief mindfulness intervention for college student binge drinkers: A pilot study. Psychology of Addictive Behaviors.

[b0475] Moeller S.J., Stoops W.W. (2015). Cocaine choice procedures in animals, humans, and treatment-seekers: Can we bridge the divide?. Pharmacology Biochemistry and Behavior.

[b0480] Mrazek M.D., Smallwood J., Schooler J.W. (2012). Mindfulness and mind-wandering: Finding convergence through opposing constructs. Emotion.

[b0485] Ortner C.N.M., Kilner S.J., Zelazo P.D. (2007). Mindfulness meditation and reduced emotional interference on a cognitive task. Motivation and Emotion.

[b0490] Paz R., Zvielli A., Goldstein P., Bernstein A. (2017). Brief mindfulness training de-couples the anxiogenic effects of distress intolerance on reactivity to and recovery from stress among deprived smokers. Behaviour Research and Therapy.

[b0495] Pilkonis P.A., Yu L., Dodds N.E., Johnston K.L., Lawrence S.M., Daley D.C. (2016). Validation of the alcohol use item banks from the Patient-Reported Outcomes Measurement Information System (PROMIS®). Drug and Alcohol Dependence.

[b0500] Ramsburg, J. T., & Youmans, R. J. (2012). Think outside the box: The effects of cognitive training on creative problem solving. In Proceedings of the Annual Meeting of the Cognitive Science Society & Natural Resources.

[b0505] Ramsburg J.T., Youmans R.J. (2014). Meditation in the higher-education classroom: Meditation training improves student knowledge retention during lectures. Mindfulness.

[b0510] Randall D.M., Cox W.M. (2001). Experimental mood inductions in persons at high and low risk for alcohol problems. The American Journal of Drug and Alcohol Abuse.

[b0515] Rejeski W.J., Thompson A., Brubaker P.H., Miller H.S. (1992). Acute exercise: Buffering psychosocial stress responses in women. Health Psychology.

[b0520] Roos C.R., Bowen S., Witkiewitz K. (2017). Baseline patterns of substance use disorder severity and depression and anxiety symptoms moderate the efficacy of mindfulness-based relapse prevention. Journal of Consulting and Clinical Psychology.

[b0525] Roos C.R., Pearson M.R., Brown D.B. (2015). Drinking motives mediate the negative associations between mindfulness facets and alcohol outcomes among college students. Psychology of Addictive Behaviors.

[b0530] Rousseau G.S., Irons J.G., Correia C.J. (2011). The reinforcing value of alcohol in a drinking to cope paradigm. Drug and Alcohol Dependence.

[b0535] Samet S., Fenton M.C., Nunes E., Greenstein E., Aharonovich E., Hasin D. (2013). Effects of independent and substance-induced major depressive disorder on remission and relapse of alcohol, cocaine and heroin dependence. Addiction (Abingdon, England).

[b0540] Sanchez A., Vazquez C., Gomez D., Joormann J. (2014). Gaze-fixation to happy faces predicts mood repair after a negative mood induction. Emotion.

[b0545] Sancho M., De Gracia M., Rodríguez R.C., Mallorquí-Bagué N., Sánchez-González J., Trujols J., Menchón J.M. (2018). Mindfulness-based interventions for the treatment of substance and behavioral addictions: A systematic review. Frontiers in psychiatry.

[b0550] Sauer S.E., Baer R.A. (2012). Ruminative and mindful self-focused attention in borderline personality disorder. Personality Disorders: Theory, Research, and Treatment.

[b0555] Schartau P.E., Dalgleish T., Dunn B.D. (2009). Seeing the bigger picture: Training in perspective broadening reduces self-reported affect and psychophysiological response to distressing films and autobiographical memories. Journal of Abnormal Psychology.

[b0560] Schwandt M.L., Cortes C.R., Kwako L.E., George D.T., Momenan R., Sinha R., Heilig M. (2016). The CRF1 antagonist verucerfont in anxious alcohol-dependent women: Translation of neuroendocrine, but not of anti-craving effects. Neuropsychopharmacology.

[b0565] Sihvola E., Rose R.J., Dick D.M., Pulkkinen L., Marttunen M., Kaprio J. (2008). Early-onset depressive disorders predict the use of addictive substances in adolescence: A prospective study of adolescent Finnish twins. Addiction.

[b0570] Singer A.R., Dobson K.S. (2007). An experimental investigation of the cognitive vulnerability to depression. Behaviour Research and Therapy.

[b0575] Singer A.R., Dobson K.S. (2009). The effect of the cognitive style of acceptance on negative mood in a recovered depressed sample. Depression and Anxiety.

[b0580] Sinha R., Fox H.C., Hong K.A., Bergquist K., Bhagwagar Z., Siedlarz K.M. (2009). Enhanced negative emotion and alcohol craving, and altered physiological responses following stress and cue exposure in alcohol dependent individuals. Neuropsychopharmacology.

[b0585] Sinha R., Fox H.C., Hong K., Hansen J., Tuit K., Kreek M. (2011). Effects of adrenal sensitivity, stress- and cue-induced craving, and anxiety on subsequent alcohol relapse and treatment outcomes. Archives of General Psychiatry.

[b0590] Smolarski K., Leone K., Robbins S.J. (2015). Reducing negative mood through drawing: Comparing venting, positive expression, and tracing. Art Therapy.

[b0595] Spanagel R., Noori H.R., Heilig M. (2014). Stress and alcohol interactions: Animal studies and clinical significance. Trends in Neurosciences.

[b0600] Spitzer R.L., Kroenke K., Williams J.B., Lowe B. (2006). A brief measure for assessing generalized anxiety disorder: The GAD-7. Archives of Internal Medicine.

[b0605] Stasiewicz P.R., Bradizza C.M., Schlauch R.C., Coffey S.F., Gulliver S.B., Gudleski G.D., Bole C.W. (2013). Affect regulation training (ART) for alcohol use disorders: Development of a novel intervention for negative affect drinkers. Journal of Substance Abuse Treatment.

[b0610] Stasiewicz P.R., Bradizza C.M., Slosman K.S. (2018). Emotion Regulation Treatment of Alcohol Use Disorders: Helping Clients Manage Negative Thoughts and Feelings.

[b0615] Tang Y.-Y., Ma Y., Wang J., Fan Y., Feng S., Lu Q., Posner M.I. (2007). Short-term meditation training improves attention and self-regulation. Proceedings of the National Academy of Sciences.

[b0620] Tang Y.-Y., Tang R., Posner M.I. (2013). Brief meditation training induces smoking reduction. Proceedings of the National Academy of Sciences.

[b0625] Tapper K. (2018). Mindfulness and craving: Effects and mechanisms. Clinical Psychology Review.

[b0630] Trask P.C., Sigmon S.T. (1999). Ruminating and distracting: The effects of sequential tasks on depressed mood. Cognitive Therapy and Research.

[b0635] Tull M.T., Bardeen J.R., DiLillo D., Messman-Moore T., Gratz K.L. (2015). A prospective investigation of emotion dysregulation as a moderator of the relation between posttraumatic stress symptoms and substance use severity. Journal of Anxiety Disorders.

[b0640] Van Dillen L.F., Heslenfeld D.J., Koole S.L. (2009). Tuning down the emotional brain: An fMRI study of the effects of cognitive load on the processing of affective images. NeuroImage.

[b0645] Van Dillen L.F., Koole S.L. (2007). Clearing the mind: A working memory model of distraction from negative mood. Emotion.

[b0650] Van Dillen L.F., Koole S.L. (2009). How automatic is “automatic vigilance”? The role of working memory in attentional interference of negative information. Cognition and Emotion.

[b0655] Vernig P.M., Orsillo S.M. (2009). Psychophysiological and self-reported emotional responding in alcohol-dependent college students: The impact of brief acceptance/mindfulness instruction. Cognitive Behaviour Therapy.

[b0660] Vernig P.M., Orsillo S.M. (2015). Drinking motives and college alcohol problems: A prospective study. Journal of Substance Use.

[b0665] Vieten C., Astin J.A., Buscemi R., Galloway G.P. (2010). Development of an acceptance-based coping intervention for alcohol dependence relapse prevention. Substance Abuse.

[b0670] Vinci C., Peltier M.R., Shah S., Kinsaul J., Waldo K., McVay M.A., Copeland A.L. (2014). Effects of a brief mindfulness intervention on negative affect and urge to drink among college student drinkers. Behavior Research and Therapy.

[b0675] Witkiewitz K., Bowen S. (2010). Depression, craving, and substance use following a randomized trial of mindfulness-based relapse prevention. Journal of Consulting and Clinical Psychology.

[b0680] Witkiewitz K., Bowen S., Douglas H., Hsu S.H. (2013). Mindfulness-based relapse prevention for substance craving. Addictive Behaviors.

[b0685] Wong K.F., Massar S.A.A., Chee M.W.L., Lim J. (2018). Towards an objective measure of mindfulness: replicating and extending the features of the breath-counting task. Mindfulness.

[b0690] Woud M.L., Becker E.S., Rinck M., Salemink E. (2015). The relationship between drinking motives and alcohol-related interpretation biases. Journal of Behavior Therapy and Experimental Psychiatry.

[b0695] Yaremko R.M., Butler M.C. (1975). Imaginal experience and attenuation of the galvanic skin response to shock. Bulletin of the Psychonomic Society.

[b0700] Yaremko R.M., Glanville B.B., Leckart B.T. (1972). Imagery-mediated habituation of the orienting reflex. Psychonomic Science.

[b0705] Zack M., Poulos C.X., Fragopoulos F., MacLeod C.M. (2003). Effects of negative and positive mood phrases on priming of alcohol words in young drinkers with high and low anxiety sensitivity. Experimental and Clinical Psychopharmacology.

[b0710] Zack M., Poulos C.X., Fragopoulos F., Woodford T.M., MacLeod C.M. (2006). Negative affect words prime beer consumption in young drinkers. Addictive Behaviors.

[b0715] Zack M., Toneatto T., MacLeod C.M. (1999). Implicit activation of alcohol concepts by negative affective cues distinguishes between problem drinkers with high and low psychiatric distress. Journal of Abnormal Psychology.

[b0720] Zgierska A., Rabago D., Zuelsdorff M., Coe C., Miller M., Fleming M. (2008). Mindfulness meditation for alcohol relapse prevention: A feasibility pilot study. Journal of Addiction Medicine.

[b0725] Zimmermann P., Wittchen H.U., Höfler M., Pfister H., Kessler R.C., Lieb R. (2003). Primary anxiety disorders and the development of subsequent alcohol use disorders: A 4-year community study of adolescents and young adults. Psychological Medicine.

