# Supplementary material for: Ultra-brief breath counting (mindfulness) training promotes recovery from stress-induced alcohol-seeking in student drinkers
Source: Addict Behav. 2020 Mar;102:106141. doi: 10.1016/j.addbeh.2019.106141 (PMC6959458; doi:10.1016/j.addbeh.2019.106141)
Supplement: Supplementary data 1 [file mmc1.docx]

# Breath counting script

In this exercise, you will learn a mediation technique that has been practiced for thousands of years.

You will learn to count your breaths, as you breathe at your own pace.

Counting your breaths as they occur is a natural way to switch your attention away from unwanted distractions in life.

This training exercise will last for only 6 minutes.

[**Relaxation – Settling in**]: To begin with… if it’s comfortable to do so, please sit with your back relatively straight, your legs uncrossed, your head and neck comfortably balanced on your shoulders and your hands resting in your lap or on the table in a comfortable way. And inviting your shoulders to be relaxed as best you can. Letting your attention settle into your body,

And now closing your eyes will help you to become aware of the experience of breathing. And as we go through this exercise, please keep your eyes closed if you find it comfortable. Start by bringing your attention to the sensations in the chest, as they expand with the in-breath, and contract with the out-breath. Try placing a hand on your chest, over your heart, to help you notice the movements here as you breathe in, and breathe out. Now switch your attention to the passage of air in and out of your nose or mouth. You may notice a difference in temperature between the in breath and the out breath.

During this practice it’s best to choose one particular aspect of the breath, for example, the movement of the chest or the sensations of the passage of air through your nose or mouth. Use these sensations as the focus of your attention.

Inevitably your mind will wander, perhaps to other sensations, perhaps to thoughts about the future or past. It is normal for your mind to wander. Simply bring your attention back to the sensations of breathing.

[**pause 5 seconds**]

[**Counting Instructions**]: You will now learn to count your breaths, to help you remain focused on the sensations of breathing.

All you have to do is count each out-breath as it finishes, counting up to ten breaths, and then start again from one.

As you come to the end of an out-breath count one. Say the number softly to yourself so that you can focus on the sensations of breathing.

After your breath moves in and out again, say two, at the end of the out-breath.

Continue, counting each out-breath, counting up to ten.

When you reach ten, start again.

Try not to control your breathing. Just allow yourself to breathe normally.

Try to keep your attention focused on the sensations of breathing.

Please practice this on your own for a while.

[**Silence 50 seconds**]

REMINDER INSTRUCTIONS **1**

1. Gently count each out-breath from one to ten.
2. Allow yourself to breathe normally.
3. Bring your attention back to the sensations of breathing if your mind wanders.

[**Silence 50 seconds**]

REMINDER INSTRUCTIONS **2**

1. Gently count each out-breath from one to ten.
2. Allow yourself to breathe normally.
3. Bring your attention back to the sensations of breathing if your mind wanders.

[**Silence 50 seconds**]

[**Final instructions**]

And now, when you feel ready, bring your attention back into the room, and gently make the transition from this exercise to the rest of the experiment.

# Control script – extract from Bill Bryson’s A Short History of Nearly Everything

We know that in 1772, at Maskelyne’s behest, he accepted the commission to find a suitable mountain for the gravitational deflection experiment, at length reporting back that the mountain they needed was in the central Scottish Highlands, just above Loch Tay, and was called Schiehallion. Nothing, however, would induce him to spend a summer surveying it.

So, for four months in the summer of 1774, Maskelyne lived in a tent in a remote Scottish glen and spent his days directing a team of surveyors, who took hundreds of measurements from every possible position. To find the mass of the mountain from all these numbers required a great deal of tedious calculating, for which a mathematician named Charles Hutton was engaged.

Hutton calculated the mass of the earth at 5,000 million million tons, from which could reasonably be deduced the masses of all the other major bodies in the solar system, including the Sun. So from this one experiment we learned the masses of the Earth, the Sun, the Moon, and the other planets and their moons – not bad for a summer’s work.

Not everyone was satisfied with the results, however. The shortcoming of the Schiehallion experiment was that it was not possible to get a truly accurate figure without knowing the actual density of the mountain. For convenience, Hutton had assumed that the mountain had the same density as ordinary stone, but this was little more than an educated guess.

One improbable-seeming person who turned his mind to the matter was a country parson named John Mitchell who, despite his humble situation, was one of the great scientific thinkers of the eighteenth century and much esteemed for it.

Among a great deal else, he perceived the wavelike nature of earthquakes and, quite extraordinarily, envisioned the possibility of black holes two hundred years before anyone else. But of all that Mitchell accomplished, nothing had greater impact than a machine he designed and built for measuring the mass of the Earth. Unfortunately, he died before he could conduct the experiments, and the necessary equipment was passed on to a brilliant but magnificently retiring London scientist named Henry Cavendish.

Cavendish is a book in himself. Born into a life of sumptuous privilege – his grandfathers were dukes, respectively, of Devonshire and Kent – he was the most gifted English scientist of his age, but also the strangest. He suffered, in the words of one of his few biographers, from shyness to a ‘degree bordering on disease’.

Although he did sometimes venture into society – he was particularly devoted to the weekly scientific soirees of the great naturalist Sir Joseph Banks – it was always made clear to the other guests that Cavendish was on no account to be approached or even looked at.

His wealth and solitary inclinations allowed him to turn his house in Clapham into a large laboratory. Cavendish was the first person to isolate hydrogen and the first to combine hydrogen and oxygen to form water. His experiments with electrical conductivity were a century ahead of their time, but unfortunately the greater part of what he did wasn’t known until the late nineteenth century, when the Cambridge physicist James Clerk Maxwell took on the task of editing Cavendish’s papers, by which time credit had nearly always been given to others.

But our interest here is in Cavendish’s last known experiment when, in the late summer of 1797, he turned his attention to the crates of equipment that had been left to him, evidently out of simple scientific respect, by John Mitchell. When assembled, Mitchell’s apparatus looked like nothing so much as an 18th century version of a nautilus weight training machine. Not a whisper of disturbance could be allowed into the room containing the apparatus. The work was incredibly exacting, and involved 17 delicate interconnected measurements, which together took nearly a year to complete. When at last he had finished his calculations, Cavendish announced that the Earth weighed a little over 6 billion trillion metric tons, to use the modern measure. A metric ton is one thousand kilograms or 2,205 pounds.

Today, scientists have at their disposal machines so precise that they can detect the weight of a single bacterium. But they have not significantly improved on Cavendish’s measurements of 1797. Interestingly, all of this merely confirmed estimates made by Newton, a hundred and ten years before Cavendish, without any experimental evidence at all.

So, by the late eighteenth century scientists knew very precisely the shape and dimensions of the Earth, and its distance from the Sun and planets. And now Cavendish had given them its weight. So you might think that determining the age of the Earth would be relatively straightforward. After all, the necessary materials were literally at their feet. But no, human beings would split the atom and invent television, nylon, and instant coffee before they could figure out the age of their own planet.

To understand why we must travel north to Scotland, and begin with a brilliant genial man of whom few have ever heard, who had just invented a new science called Geology.

At just the time that Henry Cavendish was completing his experiments in London, four hundred miles away in Edinburgh another kind of concluding moment was about to take place with the death of James Hutton. This was bad news for Hutton, of course, but good news for science as it cleared the way for a man named James Playfair to rewrite Hutton’s work without fear of embarrassment.

Hutton was without rival when it came to understanding the mysterious slow processes that shaped the Earth.
